# The Effect of Longer-Term and Exclusive Breastfeeding Promotion on Visual Outcome in Adolescence

**DOI:** 10.1167/iovs.17-23211

**Published:** 2018-06

**Authors:** Christopher G. Owen, Emily Oken, Alicja R. Rudnicka, Rita Patel, Jennifer Thompson, Sheryl L. Rifas-Shiman, Konstatin Vilchuck, Natalia Bogdanovich, Mikhail Hameza, Michael S. Kramer, Richard M. Martin

**Affiliations:** 1 Population Health Research Institute, St. George's, University of London, Cranmer Terrace, London, United Kingdom; 2 Department of Population Medicine, Harvard Medical School and Harvard Pilgrim Health Care Institute, Boston, Massachusetts, United States; 3 Bristol Medical School: Population Health Sciences, University of Bristol, Bristol, United Kingdom; 4 National Research and Applied Medicine Mother and Child Centre, Minsk, Republic of Belarus; 5 Departments of Pediatrics and of Epidemiology, Biostatistics and Occupational Health, McGill University Faculty of Medicine, Montreal, Canada; 6 University Hospitals Bristol NHS Foundation Trust and University of Bristol National Institute for Health Research Bristol Biomedical Research Centre, Bristol, United Kingdom; 7 Medical Research Council Integrative Epidemiology Unit at the University of Bristol, Bristol, United Kingdom

**Keywords:** breastfeeding, vision, presumed myopia, childhood

## Abstract

**Purpose:**

Breastfeeding may influence early visual development. We examined whether an intervention to promote increased duration and exclusivity of breastfeeding improves visual outcomes at 16 years of age.

**Methods:**

Follow-up of a cluster-randomized trial in 31 Belarusian maternity hospitals/polyclinics randomized to receive a breastfeeding promotion intervention, or usual care, where 46% vs. 3% were exclusively breastfed at 3 months respectively. Low vision in either eye was defined as unaided logMAR vision of ≥0.3 or worse (equivalent to Snellen 20/40) and was used as the primary outcome. Open-field autorefraction in a subset (*n* = 963) suggested that 84% of those with low vision were myopic. Primary analysis was based on modified intention-to-treat, accounting for clustering within hospitals/clinics. Observational analyses also examined the effect of breastfeeding duration and exclusivity, as well as other sociodemographic and environmental determinants of low vision.

**Results:**

A total of 13,392 of 17,046 (79%) participants were followed up at 16 years. Low vision prevalence was 19.6% (95% confidence interval [CI]: 17.5, 22.0%) in the experimental group versus 21.6% (19.5, 23.8%) in the control group. Cluster-adjusted odds ratio (OR) of low vision associated with the intervention was 0.92 (95% CI: 0.73, 1.16); 0.88 (95% CI: 0.74, 1.05) after adjustment for parental and early life factors. In observational analyses, breastfeeding duration and exclusivity had no significant effect on low vision. However, maternal age at birth (OR: 1.13, 95% CI: 1.07, 1.14/5-year increase) and urban versus rural residence were associated with increased risk of low vision. Lower parental education, number of older siblings was associated with a lower risk of low vision; boys had lower risk compared with girls (0.64, 95% CI: 0.59,0.70).

**Conclusions:**

Exclusive breastfeeding promotion had no significant effect on visual outcomes in this study, but other environmental factors showed strong associations. (ClinicalTrials.gov number, NCT01561612.)

Myopia is a leading cause of preventable blindness in developing countries^1^ and of correctable visual loss in the developed world.^2–6^ Myopia often begins in early life. Current global estimates suggest that 310 million children are myopic.^7^ Rapid increases in childhood myopia prevalence over time, particularly in East Asia, together with higher risk of myopia in urban settings, suggests that environmental factors play an important etiological role. Recent interest has therefore focused on the early origins of myopia and whether exposures in early life pattern myopic risk. Vision is a neurocognitive outcome that is immature at birth and programmed by visual stimuli and nutrition in early life.^8^ Inadequate infant nutrition may alter visual development,^9^ and the absence of a clear retinal image may lead to myopia.^10^ Some evidence suggests that breastfeeding promotes visual development, and hence less susceptibility to ametropia (refractive error),^9,11,12^ findings that have been attributed to the long-chain n-3 polyunsaturated fatty acids (LCPUFAs) present in breast milk. However, more recent survey evidence has been less supportive,^13^ and although LCPUFAs occur in high concentrations in retinal photoreceptors, trials comparing LCPUFA supplemented with unsupplemented formula have yielded equivocal results.^14,15^ Inconsistencies may be due to differences in LCPUFA exposure, statistical power or, in observational studies, the degree of adjustment for confounders.^9,11^ Opportunities for experimental studies to investigate the role of infant feeding on visual development are limited, given that it is infeasible and unethical to randomize healthy term infants to different feeding practices.

The Promotion of Breastfeeding Intervention Trial (PROBIT) is a large cluster-randomized controlled trial of breastfeeding promotion carried out in the Republic of Belarus, which achieved substantial differences in the exclusivity and duration of breastfeeding in 17,046 infants randomized to receive the intervention versus usual care.^16^ Follow-up of these participants allows a test of the long-term causal effects of breastfeeding on reduced unaided vision,^17^ which may provide a marker of myopia in this age group.^5^ We carried out an intention-to-treat analysis in PROBIT to provide experimental evidence on whether increased breastfeeding duration and exclusivity improves visual outcome in adolescence.

## Patients and Methods

Details of the design and phases of follow-up in PROBIT have been published elsewhere.^16,18–20^ In brief, PROBIT is a randomized controlled trial of breastfeeding promotion, which recruited 17,046 mother-infant pairs who had initiated breast feeding. The trial was carried out in the Republic of Belarus at a time when few mothers breastfed exclusively, and half discontinued breastfeeding completely by 3 months postpartum. Hence a breastfeeding promotion trial provided great potential to increase exclusivity and duration of breastfeeding.^18^ Units of randomization for the study were maternity hospitals and their associated polyclinics. These units were randomly assigned to a control group, consisting of continuation of breastfeeding practices and policies in effect at the time of randomization, or an experimental intervention based on the Baby-Friendly Hospital Initiative developed by the World Health Organization and United Nations Children's Fund to promote and support breastfeeding, particularly among mothers choosing to initiate breastfeeding.^16^ The trial results are based on 17,046 healthy breastfed infants from 31 units, born at term (≥37 completed weeks gestation) in 1996/97 and enrolled during their postpartum stay. Trial inclusion criteria required infants to be healthy, singleton, birthweight ≥2.5 kg, Apgar score ≥5 at 5 minutes, and mothers to have initiated breastfeeding and no condition known to impede breastfeeding.^16^

### Follow-Up

The mother-infant pairs were followed up frequently for 12 months from the time of birth. Additional follow-up was carried out between 2002 and 2005, when the children were aged 6.5 years, and again between 2008 and 2010 when the children were aged 11.5 years. This paper focuses on an additional follow-up at 16 years, when lung function and neurocognitive outcomes (including vision) were assessed.^21^ The 16-year follow-up was approved by the Belarusian Ministry of Health. Ethical approval was obtained prospectively from the McGill University Health Centre Research Ethics board, the Human Subjects Committee at Harvard Pilgrim Health Care, and the Avon Longitudinal Study or Parents and Children Law and Ethics Committee. Parents of guardians provided informed consent and children gave written assent. The research adhered to the tenets of the Declaration of Helsinki.

Trained pediatricians performed in-person research assessments with children at 31 polyclinics, between September 2012 and July 2015. Training included a 2-day initial workshop, tutoring, and practical sessions, with retraining every 6 months. Quality assurance was ensured by ongoing data monitoring, as described previously.^22^ One polyclinic was excluded due to deviations from the study protocol.

### Vision Assessment

Unaided distance vision was measured in each eye at 3 m using LogMAR acuity charts with Cyrillic type face (Keeler Ltd., Windsor, UK); tests were repeated at 1 minute if the largest letters (line 1) could not be seen. Visual acuity was measured in each eye with current spectacle correction if present. Vision tests were repeated with a pin hole if vision was line 6 or worse (logMAR ≥0.3, equivalent to Snellen ≥6/12, ≥20/40).

### Vision Outcomes

Low vision was defined as unaided vision of line 6 (logMAR 0.3, equivalent to Snellen 6/12, 20/40) or worse in either eye. The test performance of this measure as a proxy for myopia has previously been shown to be high in this age group.^5^ We also validated the test in a sub-sample (children at one of the largest polyclinics) using open field autorefractometry. Secondary outcomes included anisopia (unequal vision), defined as a difference in unaided vision of 0.2 logMAR acuity or more between eyes, and normal vision, defined as unaided vision of line 9 or better (logMAR 0, equivalent to 6/6, 20/20) in either eye (with and without use of spectacles or a pinhole).

### Validation of Vision Outcome

Five measures of ocular refraction in each eye were obtained without cycloplegia using an open-field autorefractor (WAM-5500, Grand Seiko Co., Ltd, Japan) in the subsample of all children seen at a single large polyclinic (∼8% of the total children examined). Each child was seated with the head positioned using chin and forehead rests, with eyes aligned with the eye mark, while observing a nonaccommodative target (red Maltese cross) at 3 m through the viewing window. The accuracy and vertex distance of the instrument were set to the default settings of 0.25 diopters (D) and 12 mm, respectively. Given the use of autorefraction without cycloplegia and the distance of the accommodative target (3 m) we defined myopia as a spherical equivalent refraction (SER) of −1.00 D or worse in either eye (using autorefractor measurements in negative cylinder form), which is more conservative compared to definitions used previously with SER of −0.50 D or worse.^23,24^ The maximum positive (least negative) of five readings in each eye was used; cycloplegia was avoided to maximize participation.

### Baseline Covariates

Data on urban and rural location of polyclinic, maternal age at birth, maternal smoking during pregnancy, maternal and parental education, child's sex, birthweight, and number of older siblings were recorded from earlier phases of the study.^16^ The parent or guardian provided information on current family size.

### Statistical Analysis

Analyses were carried out using statistical software (STATA version 13; StataCorp, College Station, TX, USA). The distributions of covariates in children in the intervention and control groups were compared. The primary outcome was logMAR acuity. However, the distribution of logMAR acuity showed a highly positive skew, heavily truncated at high levels of acuity, which could not be readily transformed to normalize the distribution. Hence, logMAR acuity was dichotomized, with low vision defined as unaided vision of Line 6 (logMAR 0.3, equivalent to Snellen 6/12, 20/40) or worse in either eye.^24,25^ Other dichotomized outcomes included anisopia, and normal vision (defined above). Intra-class correlation coefficients (ICC) for these outcomes were calculated to examine the degree of clustering. The primary intention-to-treat analysis used multiple variable logistic regression to obtain odds ratios for vision outcomes by intervention groups, accounting for clustering of children within clinics (using meqrlogit, multilevel mixed-effects logistic regression). Consistency of effects by sex were examined by multiplicative interaction terms. Secondary analyses additionally adjusted for stratum-level variables (urban versus rural and residence in West versus East Belarus), and for early-life factors (birthweight, birth order, family size) and parental factors (maternal age at birth, maternal and paternal education), which, given the small number of clusters randomized, could theoretically confound the effect estimates observed.

Comparisons between the intervention and experimental groups were based on children with observed vision outcomes without imputation, owing to the very high participation rates in this cohort. As intention-to-treat analysis systematically underestimates the effect of actual breastfeeding duration and exclusivity on vision outcome, owing to substantial overlap in feeding practices between the randomized groups, we applied instrumental variable methods to account for this overlap.^26^ We used randomization status as the instrument, since it is independent of any confounders of the exposure-outcome relationship and is related to the outcomes only via its effect on breastfeeding, which we dichotomized as ≥3 vs. <3 months of exclusive breastfeeding. We performed instrumental variable estimation of the low vision dichotomous outcome using probit regression (ivprobit command in STATA) with robust standard error estimation to allow for clustering by hospital. We did not carry out multiple imputation for missing outcome data given the high level of follow-up and lack of vision measurement in earlier phases that we could use to impute vision at 16 years.

We also used multivariable logistic regression in observational analyses (i.e., disregarding randomization status) to examine the associations of other infant (birthweight, breastfeeding duration and exclusivity, maternal age and smoking at birth), child (sex, number of older/younger siblings) and familial (maternal/paternal education, urban-rural East-West residence) factors with vision outcomes. We also carried out an observational analysis in the sub-set who underwent autorefraction to examine the effect of breast feeding duration and exclusivity on measured myopia (defined as a SER of −1.00 D or worse in either eye) and astigmatism (defined as −1.00 DC of astigmatism or more in either eye). We categorized durations of any breastfeeding and exclusive breastfeeding as less than 3 months (reference standard), 3 months to less than 6 months, and 6 months or more. Odds ratios allowing for the random effect of hospital only and multivariable-adjusted odds ratios are presented.

## Results

**Figure 1:**
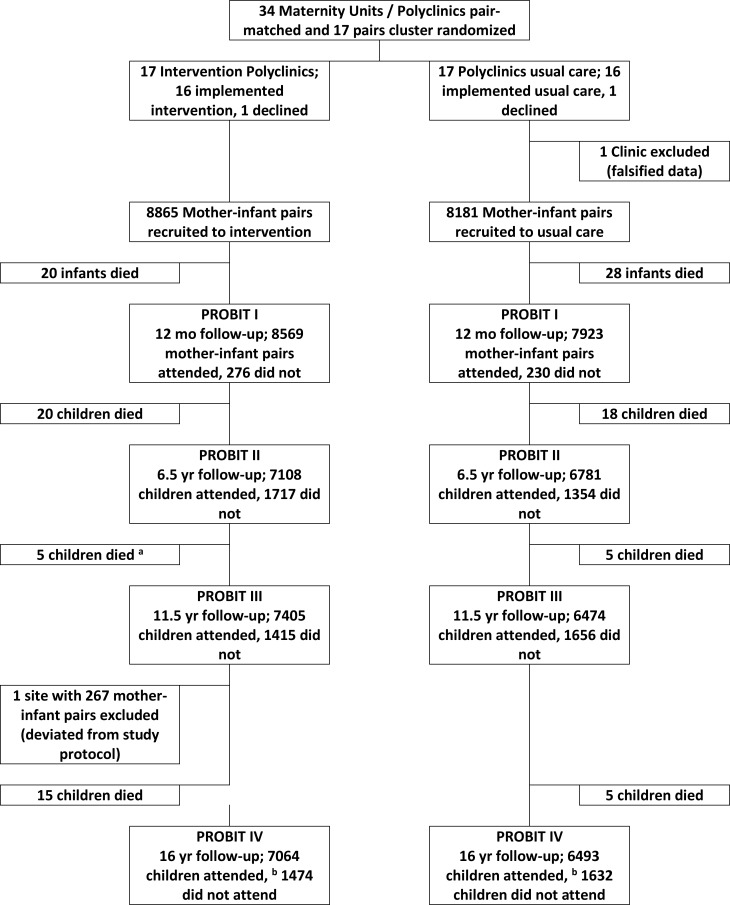
Flow diagram of clusters and individuals examined at PROBIT recruitment and follow phases I, II, III, and IV at 15 years of age. ^a^ During PROBIT III, six deaths were reported in the intervention arm. Data checking during PROBIT IV found one of these children had been incorrectly reported as deceased and data were amended. ^b^ Of the 13,557 seen at PROBIT IV, 12,072 were seen at both PROBIT II & III; 274 were not seen at either PROBIT II & III; 449 were seen at PROBIT II but not seen at III; and 762 were seen at PROBIT III but not seen at II.

Figure 1 shows the numbers of infant and mother pairs randomized to breastfeeding promotion versus usual care in the 31 Belarusian recruited polyclinics that participated in successive phases of the PROBIT study. A total of 13,557 children were examined at a median age of 16.1 years (SD: 0.5; interquartile range: 15.8, 16.4 years), representing 79.5% of those originally randomized (Fig. 1). Of the 3489 children randomized but not followed up at 16 years, 116 had died since randomization, 2674 were lost to follow-up, 267 were excluded from one clinic that deviated from the study protocol, and 432 were unable or unwilling to come for their visit (Fig. 1). The primary vision outcome was available for 99% of participants who took part (*n*/*N* = 13,392/13,557); 6969 in the intervention group and 6423 in the control group. Follow-up rates were similar overall in the experimental (79.7%) and control (79.3%) polyclinics.

Table 1 gives the baseline and follow-up characteristics of intervention and control groups amongst those with visual outcome, which were similar. In the follow-up at 16 years of age, sociodemographic characteristics were similar between the two groups in all respects, except for over-representation of urban households in Western Belarus among the intervention group compared to controls (Table 1). As previously reported, the intervention increased the duration of exclusive breastfeeding,^16^ based on criteria defined by the Word Health Organization,^27^ compared to the control group. Among those with visual outcome, 45% in the intervention group versus 6.9% in the control group were exclusively breastfed for 3 months or more (Table 1).

**Table 1:**
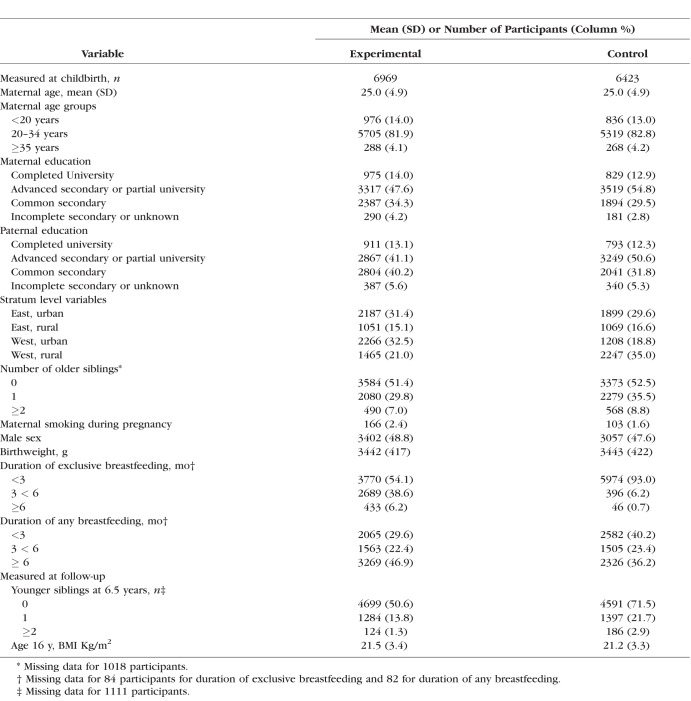
Baseline and Follow-Up Characteristics in the Intervention and Control Groups With the Primary Vision Outcome

Table 2 shows the prevalence of low vision, anisopia and normal vision according to randomized group. Among children allocated to the intervention, the prevalence of low vision was 19.6%, and among those allocated to control was 21.6%, yielding a cluster-adjusted odds ratio of 0.92 (95% CI: 0.73, 1.16), which was 0.88 (95% CI: 0.74, 1.05) after additional adjustment for family size, birth order, birthweight, maternal age at birth, parental education, and urban versus rural residence. Effect sizes were similar for boys and girls with no evidence of modification by sex (all interaction *P* values > 0.12). Corresponding prevalences of anisopia were 7.0% and 8.1%, respectively, yielding a cluster-adjusted OR of 0.84 (95% CI: 0.66, 1.08) and a fully-adjusted OR of 0.86 (95% CI: 0.66, 1.10). The prevalence of normal vision without optical correction was 72.7% among children allocated to the intervention and 70.5% among controls; the resulting odds ratio was 1.21 (95% CI: 0.96, 1.52) after adjustment. Table 2 also provides data for normal vision after allowing use of spectacle correction or pinhole to achieve the best possible acuity, which was reassuringly similar (cluster-adjusted odds of 1.20, 95% CI: 0.78, 1.82; 1.31, 95% CI: 0.93, 1.83 after adjustment).

**Table 2:**
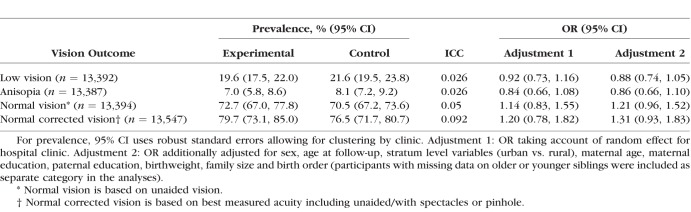
Prevalence of Low Vision and Intention to Treat Analysis (Without Imputation) Showing the OR Between Intervention and Control Group

**Figure 2:**
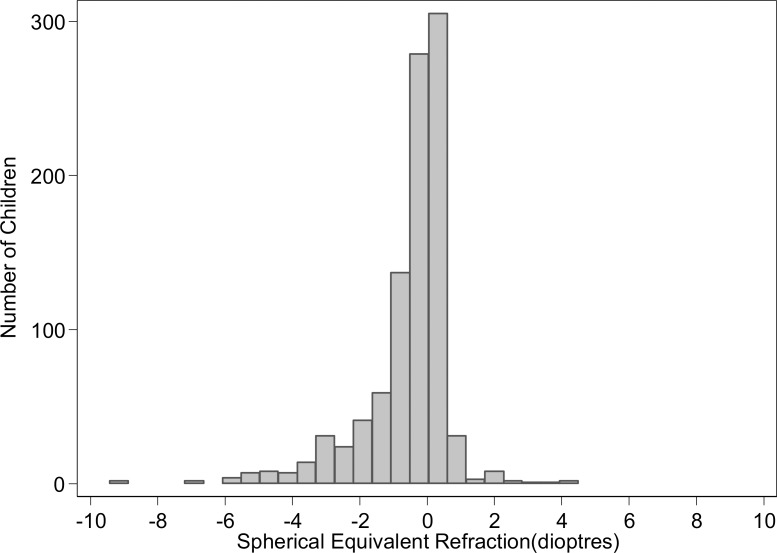
Distribution of spherical equivalent refraction in the subset who underwent autorefraction without cycloplegia (n = 963).

In analyses of the subsample with open field autorefraction (*n* = 963), 21.6% (95% CI: 19.1, 24.3%) were defined as myopic. Figure 2 shows the distribution of spherical equivalent refraction in the subsample. Of those classified with low vision, 84% had a spherical equivalent refraction of −1.00 D or less, while 4.2% of those classified with normal vision had a myopic refractive error. Because autorefraction was limited to one polyclinic where all children were randomized to the intervention, we were unable to examine whether refractive findings differed by randomized group.

Repeat measures of vision in 124 children showed that there was a high correlation with repeated unaided logMAR acuity (Spearman correlation 0.80 in right eyes and 0.83 in left eyes) and there was no statistical evidence of discordance in those classified as having low vision or not (*P* > 0.10). Repeated measures of autorefraction in 30 children showed that spherical equivalent refraction was also highly correlated (0.87 in right eyes and 0.75 in left eyes) and on average differences were less than 0.1 D.

Given the small and nonsignificant effects of the intervention on low vision, we also examined observational associations between vision and infant feeding, as well as other infant, child, familial and sociodemographic factors. Table 3 shows odds ratios adjusted for random effect of clinic only (model 1) and fully adjusted odds ratios (model 2). Infant feeding showed no consistent association with low vision in observational analyses for duration of either exclusive or any breastfeeding (Table 3).

**Table 3:**
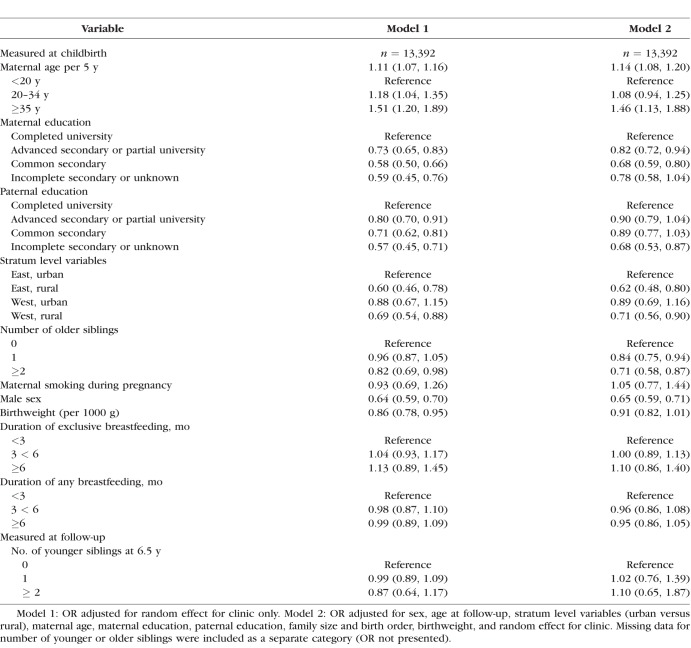
Observational Associations Between Familial and Childhood Factors With Low Vision

Results of instrumental variable analyses, which provide estimates of the unbiased associations of exclusive breastfeeding for 3 months or longer versus less than 3 months (and are therefore directly comparable to estimates from observational studies), indicated that increased duration and exclusivity of breastfeeding provided no important beneficial effects on the vision outcomes examined. Moreover, no association was observed between duration of any or exclusive breastfeeding and measured myopia (or astigmatism) in the subset who underwent autorefraction (Supplementary Table S1).

Further observational analyses showed that boys were less likely to have low vision (OR: 0.64, 95% CI: 0.59, 0.70) than girls. Increased maternal age at birth showed a strong positive association with low vision. Decreasing maternal and paternal education showed strong graded associations with low vision. Urban-rural residence showed strong associations with low vision; children living in rural settings were less likely to have low vision compared to those living in urban environments. A strong inverse association between the number of older siblings and low vision was observed. Low vision showed no consistent association with birthweight, maternal smoking during pregnancy or the number of younger siblings. Corresponding associations with secondary outcomes, including anisopia and normal vision are shown in Supplementary Table S2. Boys were less likely to have anisopia (0.70, 95% CI: 0.61, 0.80) than girls and were 50% more likely to have normal vision (1.50, 95% CI: 1.39, 1.62). Increased maternal age showed no clear trend with anisopia, but an inverse association with normal vision. Decreasing parental education showed a modest graded association with anisopia and commensurate inverse associations with normal vision. Children living in rural settings were more likely to have normal vision compared to those living in urban environments. A positive association between the number of older siblings and normal vision was observed, with no evidence of an association with anisopia. As with low vision, secondary vision outcomes showed no consistent association with birthweight, maternal smoking during pregnancy or the number of younger siblings.

## Discussion

### Main Findings

Whether infant feeding, breastfeeding in particular affects visual development in early life remains controversial. We examined the effect of a breastfeeding promotion intervention on visual development in adolescence; the intervention had a small but nonsignificant beneficial effect on a number of vision outcomes; nor was there any association with breastfeeding duration or exclusivity in observational analyses. In contrast, other observational findings show that factors such as sex, parental education and place of residence have far greater influences on visual outcome in early life, suggesting that early visual development might be more strongly patterned by other (besides infant feeding) potentially modifiable environmental factors.

### Relation to Earlier Studies

Several previous observational studies have suggested better visual outcomes in infancy and early childhood among children who were breastfed compared to those formula-fed.^9,11,28,29^ However, not all such studies have been as supportive; some suggest no effect^13^ or associations that might be partially or wholly explained by residual confounding.^25^ Our finding of similar levels of low vision amongst children from maternity hospitals and clinics randomized to breastfeeding promotion or continued standard practice suggests that longer duration and more exclusive breastfeeding has little effect in patterning vision outcomes into late childhood.

An effect of breastfeeding is biologically plausible, given the abundance of n-3 LCPUFAs in breast milk, which are needed for early neurocognitive development.^30^ In utero exposure to LCPUFAs is particularly important in the last trimester of pregnancy.^31^ This has been hypothesized as the reason why randomized clinical trials of LCPUFA interventions in preterm infants have been more likely to show beneficial effects, compared to interventions in healthy, full-term infants.^30^ However, beneficial effects that have been observed are modest at best and do not appear to persist.^30^

The effects of infant feeding in the present study were small and dwarfed by observational findings for other sociodemographic and environmental factors. The sex effect observed (girls had worse vision outcome than boys) is congruent with previous findings showing higher levels of myopia in girls, which appears to emerge in adolescence.^7,25^ These sex differences have been attributed to a greater emphasis on education, prolonged near vision activities and stronger academic performance in girls compared to boys.^32^ The relationship of parental education with early visual development we observed is coherent with trends in myopia observed in European populations overtime^33^ and with other findings observed previously.^25^ Moreover, the association we observed with urban residence, whereby children from rural environments were less likely to have low vision compared to those living in urban environments, provides strong evidence of environmental patterning of early visual development. Children living in more congested environments, with greater emphasis on education and related near vision activities, have worse vision outcomes. However, the magnitude of the difference we observed between urban and rural residence is less than that previously reported in other populations,^7^ which has been attributed to smaller differences in living conditions among populations of European ancestry.^7^ Maternal age at birth is another factor strongly related to education, since, more educated mothers tend to delay their first birth. Maternal age also showed a strong graded association with low vision. Findings from our study also confirm the potentially beneficial effect of increasing numbers of older siblings on low vision.^34^

In our study, maternal smoking during pregnancy and birthweight showed no consistent association with low vision. Indicators of fetal growth, including birth weight, length, and head circumference have been associated with refractive error and ocular biometric measures in young children in other studies,^35,36^ and some evidence suggests the association between birthweight and reduced vision might persist into adulthood.^37^ However, others have been more equivocal about the role of birthweight and childhood growth on vision outcome in later life,^38^ particularly in adolescence.^25^

### Strengths and Limitations

PROBIT offers a unique opportunity to examine the effect of breast feeding duration and exclusivity on health outcomes. The procedure of randomization, which occurred early in life without the mother's choice or knowledge of health outcome, succeeded in achieving two highly comparable groups, wherein intention-to-treat analysis demonstrates the unconfounded effect of a marked difference in exclusive breastfeeding duration (43% vs. 6% exclusively breastfed at 3 months, 7.9% and 0.6% at 6 months) on vision and other health related outcomes.^19,39^ High response and participation rates (consistently 80% or above at all follow-up phases)^18^ minimize the risk of selection bias in comparisons between intervention groups. The large number of participants improves statistical power to detect a true association, if one exists.

However, PROBIT is a randomized trial of breastfeeding promotion, with considerable overlap in breastfeeding durations, and hence does not allow experimental comparisons between breastfeeding and non-breastfeeding groups. Such interventions are limited, given that it is infeasible and unethical to randomize healthy infants to different feeding practices. The study population is well-nourished and well-provided with healthcare services and child immunization programs. Hence, the lack of a strong association between intervention groups may not be generalizable to less privileged/well-nourished populations. A further potential limitation is the use of vision cut-offs as a proxy for refractive status, which may have also weakened the presence of any underlying association, if an association truly exists. However, the detection rate of myopia has previously been shown to be reliable in this age group^40^ and was validated in a large sub-sample who underwent open-field autorefraction (*n* = 963). While cycloplegia was avoided to encourage participation, this may have resulted in overestimation of myopia and underestimation of hyperopia in this study population. However, overestimation of myopia is less likely in older children, and levels of presumed myopia (i.e., low vision) were consistent with recent estimates for white European children of this age: 20.6% (95% CI: 19.0%–22.2%) in PROBIT as a whole, and 21.6% (95% CI: 19.1, 24.3%) in the PROBIT subsample with autorefraction versus 16.7% (95% CI: 10.6%–24.5%) in white Europeans aged 15 years (although estimates at 16 years are likely to be marginally higher).^7^ It is noteworthy that any potential overestimation should be equally represented in both the intervention and control groups and is therefore unlikely to have biased the associations observed.^41^ Moreover, consistency of effects with secondary vision outcomes, including anisopia and normal vision, and with other sociodemographic variables further supports the validity of the methodology used and associated findings. Associations with sociodemographic variables are consistent with earlier findings in differing geographical locations, time periods and study designs, and collectively this accumulation of evidence provides support for causal associations.

### Conclusions

An intervention to promote prolonged duration of exclusive breastfeeding has no significant effect on vision, suggesting that modifying infant feeding is unlikely to have an appreciable impact on refractive error amongst well-nourished populations. While urbanicity is environmentally determined, associations with parental education may reflect environmental patterning of visual behavior or genes (or a gene-environment interaction), perhaps elicited by increased education and near-vision activities which are established risk factors for myopia.^33^ However, rapid rises in myopia, and the emergence of geographic differences suggest that the environment has an important role to play in determining vision outcome, patterning the development of refractive error into later life.

## Supplementary Material

Supplement 1
