# Supplementary material for: The Effect of Longer-Term and Exclusive Breastfeeding Promotion on Visual Outcome in Adolescence
Source: Invest Ophthalmol Vis Sci. 2018 Jun;59(7):2670–8. doi: 10.1167/iovs.17-23211 (PMC5983062; doi:10.1167/iovs.17-23211)
Supplement: Supplement 1 [file iovs-59-06-43_s01.pdf]

Supplemental Table 1: Observational associations between refractive outcome and duration / exclusivity of breast feeding, in the sub-set who underwent autorefraction (n=963).

**Variable**<br>**Measured in infancy**		**Myopia**<br>N=963	**Astigmatism**<br>N=963
**Duration of exclusive breastfeeding (months)**			
	<3	reference	reference
	3 <6	1.10 (0.79, 1.51)	0.69 (0.34, 1.40)*
	>=6	1.06 (0.41, 2.74)	0.51 (0.06, 4.49)*
**Duration of any breastfeeding (months)**			
	<3	reference	reference
	3 <6	1.00 (0.64, 1.54)	1.53 (0.68, 3.46)*
	>=6	1.27 (0.89, 1.81)	0.78 (0.36, 1.72)*

Based on 40 cases of astigmatism and 209 of myopia. Odds ratio adjusted for sex, age at follow-up, stratum level variables (urban vs rural), maternal age, maternal education, paternal education, family size (*excluded for astigmatism as model did not converge) and birth order, birthweight and random effect for clinic

Supplemental Table 2: Observational associations between familial and childhood factors with all vision outcomes.

**Variable**	**Low vision**	**Anisopia**	**Normal vision***	**Normal corrected vision****
**Measured in infancy**	N=13392	N=13387	N=13394	N=13547
**Maternal age per 5 years**	1.14 (1.08, 1.20)	1.04 (0.96, 1.13)	0.89 (0.85, 0.94)	0.91 (0.86, 0.96)
<20 years	reference	reference	reference	reference
20-34 years	1.08 (0.94, 1.25)	1.16 (0.93, 1.45)	0.89 (0.78, 1.01)	0.92 (0.80, 1.06)
>=35 years	1.46 (1.13, 1.88)	1.08 (0.72, 1.61)	0.73 (0.58, 0.92)	0.75 (0.59, 0.97)
**Maternal education**				
Completed University	reference	reference	reference	reference
Advanced secondary or partial university	0.82 (0.72, 0.94)	0.96 (0.78, 1.19)	1.15 (1.01, 1.30)	1.12 (0.98, 1.29)
Common secondary	0.68 (0.59, 0.80)	0.88 (0.69, 1.12)	1.25 (1.08, 1.44)	1.15 (0.99, 1.35)
Incompleted secondary or unknown	0.78 (0.58, 1.04)	0.89 (0.57, 1.38)	1.53 (1.17, 2.01)	1.33 (0.99, 1.78)
**Paternal education**				
Completed University	reference	reference	reference	reference
Advanced secondary or partial university	0.90 (0.79, 1.04)	0.98 (0.79, 1.21)	1.06 (0.94, 1.21)	1.01 (0.88, 1.16)
Common secondary	0.89 (0.77, 1.03)	0.83 (0.66, 1.04)	1.07 (0.93, 1.23)	1.03 (0.89, 1.20)
Incompleted secondary or unknown	0.68 (0.53, 0.87)	0.91 (0.64, 1.30)	1.28 (1.03, 1.59)	1.16 (0.92, 1.46)
**Stratum level variables**				
East, urban	reference	reference	reference	reference
East, rural	0.62 (0.48, 0.80)	0.95 (0.66, 1.37)	1.80 (1.29, 2.49)	2.16 (1.33, 3.50)
West, urban	0.89 (0.69, 1.16)	1.08 (0.74, 1.57)	1.33 (0.94, 1.89)	1.48 (0.88, 2.49)
West, rural	0.71 (0.56, 0.90)	0.86 (0.62, 1.21)	1.89 (1.39, 2.56)	2.15 (1.37, 3.37)
**Number of older siblings**				
0	reference	reference	reference	reference
1	0.84 (0.75, 0.94)	1.04 (0.88, 1.23)	1.13 (1.03, 1.25)	1.10 (0.99, 1.23)
>=2	0.71 (0.58, 0.87)	1.10 (0.82, 1.46)	1.32 (1.11, 1.58)	1.18 (0.97, 1.43)
**Maternal smoking during pregnancy**	1.05 (0.77, 1.44)	1.31 (0.86, 1.99)	0.88 (0.67, 1.16)	0.79 (0.59, 1.06)
**Male sex**	0.65 (0.59, 0.71)	0.70 (0.61, 0.80)	1.50 (1.39, 1.62)	1.50 (1.38, 1.64)
**Birthweight (per 1000 g)**	0.91 (0.82, 1.01)	0.90 (0.76, 1.05)	1.08 (0.98, 1.19)	1.11 (1.00, 1.23)
**Duration of exclusive breastfeeding (months)**				
<3	reference	reference	reference	reference

3 <6	1.00 (0.89, 1.13)	1.04 (0.87, 1.25)	0.98 (0.88, 1.09)	1.05 (0.93, 1.18)
>=6	1.10 (0.86, 1.40)	1.04 (0.72, 1.52)	0.87 (0.70, 1.09)	0.80 (0.63, 1.02)
**Duration of any breastfeeding (months)**				
<3	reference	reference	reference	reference
3 <6	0.96 (0.86, 1.08)	1.10 (0.93, 1.30)	1.05 (0.95, 1.17)	1.10 (0.98, 1.24)
>=6	0.95 (0.86, 1.05)	0.94 (0.80, 1.09)	1.06 (0.97, 1.16)	1.07 (0.97, 1.18)
**Measured at follow-up**				
**No. of younger siblings at 6.5 years**				
0	reference	reference	reference	reference
1	1.02 (0.76, 1.39)	1.21 (0.79, 1.85)	1.00 (0.76, 1.32)	1.12 (0.83, 1.53)
>=2	1.10 (0.65, 1.87)	1.29 (0.62, 2.71)	1.03 (0.59, 1.81)	1.03 (0.59, 1.81)

Odds ratio adjusted for sex, age at follow-up, stratum level variables (urban vs rural), maternal age, maternal education, paternal education, family size and birth order, birthweight and random effect for clinic

Missing data for number of younger or older siblings were included as a separate category (odds ratios not presented).

*Normal vision is based on unaided vision

**Normal corrected vision is based on best measured acuity including unaided/with spectacles or pinhole
